# In Vivo Models of Diabetes: Unravelling Molecular Pathways in Metabolic and Skeletal Complications

**DOI:** 10.3390/biomedicines14010243

**Published:** 2026-01-21

**Authors:** Haryati Ahmad Hairi, Nor Hidayah Mustafa, Ahmad Nazrun Shuid, Muhammad Zulfiqah Sadikan

**Affiliations:** 1 Department of Biochemistry, Faculty of Medicine, Manipal University College Malaysia, Bukit Baru, Melaka 75150, Malaysia; haryati.hairi@manipal.edu.my; 2 Faculty of Medicine, Lincoln University College, Petaling Jaya 47301, Selangor, Malaysia; dr.nor@lincoln.edu.my; 3 Department of Pharmacology, Faculty of Medicine, Universiti Teknologi MARA (UITM), Jalan Hospital, Sungai Buloh 47000, Selangor, Malaysia; 4 Faculty of Pharmacy and Health Sciences, Universiti Kuala Lumpur Royal College of Medicine Perak, Ipoh 30450, Perak, Malaysia

**Keywords:** in vivo, diabetic osteoporosis, high-fat diet, streptozotocin, impaired insulin/IGF-I signalling

## Abstract

**Background/Objectives**: Diabetic osteoporosis (DOP) is a metabolic bone disorder marked by reduced bone mass, impaired microarchitecture and elevated fracture risk arising from type 1 and type 2 diabetes. Understanding its pathophysiology is essential for developing effective interventions. **Method**: A broad literature search of Scopus and PubMed (2015–2025) using diabetic osteoporosis-related keywords identified relevant English in vivo studies, which were screened, extracted, and narratively summarised for this review. **Results**: In vivo models, including high-fat-diet (HFD), streptozotocin (STZ) and combined HFD + STZ protocols, are widely used to investigate DOP mechanisms. HFD models mimic obesity-induced insulin resistance, chronic hyperglycaemia and low-grade inflammation, leading to suppressed osteoblast activity, enhanced osteoclastogenesis and accumulation of advanced glycation end products (AGEs). Ultimately, they compromise bone microarchitecture and mechanical strength. STZ models replicate type 1 diabetes by inducing β-cell destruction, insulin deficiency, oxidative stress, osteoblast apoptosis and inflammatory pathways promoting bone resorption. The combined HFD + STZ model integrates insulin resistance and partial β-cell dysfunction, closely reflecting type 2 diabetes pathology, including trabecular bone loss, collagen glycation and disrupted osteoblast–osteoclast signalling. Mechanistically, DOP involves impaired insulin/IGF-I signalling, AGE–RAGE interactions, oxidative stress and inflammation, resulting in diminished bone formation and quality. These models provide robust platforms for exploring molecular mechanisms and evaluating potential therapies, including Wnt pathway modulators, antioxidants and ferroptosis inhibitors. **Conclusions**: Collectively, preclinical in vivo models are indispensable for understanding DOP pathophysiology and developing strategies to mitigate diabetic bone fragility.

## 1. Introduction

Diabetes mellitus (DM) is a chronic metabolic condition caused by insufficient insulin secretion from pancreatic β cells or insulin resistance in target tissues such as skeletal muscle, adipose tissue and liver. Upon failure of these tissues to respond properly to insulin, glucose uptake declines, leading to hyperglycaemia, a condition characterised by sustained high blood sugar levels [1]. According to the International Diabetes Federation (IDF), the global diabetes prevalence is anticipated to rise from 9.3% in 2019 to 10.2% in 2030, then to 10.9% by 2045 [2]. Diabetes mellitus can be classified into three types: type I, type II and gestational diabetes. Type 1 diabetes (T1DM), also known as insulin-dependent or juvenile-onset diabetes, is caused by the autoimmune destruction of pancreatic β cells that produce insulin. Type 2 diabetes mellitus (T2DM) is the most common kind worldwide, distinguished by insulin resistance and partial insulin insufficiency [3]. Although generally associated with elderly people, T2DM has considerably increased in younger individuals (<40 years old) [4]. According to the American Diabetes Association (ADA), diagnostic criteria for T2DM in non-pregnant adults include haemoglobin A1C (HbA1C) ≥ 6.5%, fasting plasma glucose ≥ 126 mg/dL, plasma glucose ≥ 200 mg/dL two hours after a 75 g oral glucose tolerance test (OGTT), or random plasma glucose ≥ 200 mg/dL in the presence of hyperglycaemic symptoms [5]. Poor glycaemic management in T2DM is associated with poor bone remodelling, and individuals with HbA1c levels above 9.0% have a significantly increased risk of hip fractures [6].

Chronic hyperglycaemia in diabetes impairs not only glucose metabolism but also bone remodelling and repair mechanisms. In addition, prolonged exposure to high glucose and lipid levels inhibits osteoblast function, increases osteoclast activity and decreases bone mineral density, raising the risk of osteoporosis and fractures. This disorder, known as diabetic osteoporosis, is an increasingly recognised but understudied consequence of diabetes. Recent research reveals that diabetic osteoporosis varies from primary osteoporosis in terms of underlying mechanisms and clinical presentation, necessitating the development of new diagnostic, risk assessment and preventative techniques. Furthermore, diabetic patients with a history of fracture are about twice as likely to develop cardiovascular disease as those without fractures [7]. A meta-analysis conducted in China found a large prevalence of osteoporosis among people with type 2 diabetes (T2DM), affecting 44.8% of men and 37.0% of women, highlighting the severity of this condition [8]. Similarly, Schacter and Leslie (2017) found that diabetic patients have a biphasic fracture risk, which is initially lower in newly diagnosed persons but significantly higher in those with long-standing diabetes [9]. Poor glycaemic management has been repeatedly linked to increased bone fragility. For instance, research has shown that diabetic patients experience over 60% higher fracture incidence than non-diabetic individuals [10]. These findings emphasise the substantial relationship between persistent hyperglycaemia and poor bone health, highlighting the need for more mechanistic research and focused therapies in diabetic osteoporosis.

In vivo models are essential for elucidating the complex pathophysiological mechanisms connecting diabetes to bone fragility, as they allow controlled investigation of how chronic hyperglycaemia, insulin resistance, oxidative stress and inflammation impair osteoblast function, enhance osteoclast activity and disrupt bone microarchitecture. Type 1 diabetic osteoporosis is commonly induced in rodents through streptozotocin (STZ) administration, which selectively destroys pancreatic β cells, resulting in insulin deficiency and subsequent bone deterioration. For type 2 diabetic osteoporosis, high-fat-diet-induced obesity combined with low-dose STZ is widely used to replicate insulin resistance and chronic hyperglycemia, making it a commonly employed model. Other available models include genetically modified rodents such as db/db or ob/ob mice, as well as ZDF rats (Zucker Diabetic Fatty rat) and NOD mice (Non-Obese Diabetic mouse), which are occasionally used to study specific mechanisms of bone loss in diabetes [11,12].

Commonly employed models, such as high-fat-diet (HFD)-induced diabetes, streptozotocin (STZ)-induced diabetes and combined HFD + STZ protocols, provide mechanistic insights that cannot be obtained by clinical investigations alone. These preclinical models also enable the controlled, time-dependent assessment of bone metabolism, underlying biological mechanisms and the testing of novel treatment approaches. Understanding these mechanisms through in vivo models is essential for identifying key molecular pathways, validating pharmacological targets and developing effective strategies to prevent and treat diabetic osteoporosis. Accordingly, this review summarises and critically evaluates high-fat-diet and streptozotocin-induced preclinical diabetes models used to investigate metabolic and skeletal abnormalities, which then emphasises their relevance, underlying mechanisms and translational potential in elucidating diabetic osteoporosis and related bone disorders. To achieve this, a comprehensive search was performed in Scopus and PubMed for studies published between 2015 and 2025, using the search string (“in vivo” OR “diabetic mice”) AND (“diabetic osteoporosis”). All retrieved articles were screened, extracted and synthesised narratively, with only English-language publications included.

## 2. In Vivo Model of Diabetic Osteoporosis

### 2.1. High-Fat-Diet (HFD) Model of Diabetic Osteoporosis

HFD consumption in rodent models consistently results in significant skeletal deterioration, particularly affecting the trabecular bone (Table 1). Micro-computed tomography (micro-CT) (Micro-CT) analyses across multiple studies revealed marked reductions in bone volume/total volume (BV/TV), bone mineral density (BMD), trabecular number (Tb.N), trabecular thickness (Tb.Th) and connectivity density (Conn.D), alongside increased trabecular separation (Tb.Sp), which reflect a fragile and disorganised bone structure. These structural deficits are accompanied by reduced mechanical strength, including lower initiation and maximal toughness, indicating compromised bone quality even in cases where mineral density may appear elevated (Table 1). This model is widely used as a reference in vivo diabetic studies, particularly those investigating type 2 diabetes and obesity-induced metabolic dysfunction. The obese phenotype, chronic hyperglycaemia (blood glucose > 250 mg/dL) and typical diabetic symptoms, such as polyuria and polydipsia, all suggest that the HFD animals had acquired diabetes [13,14,15,16]. Obesity, chronic hyperglycaemia and overt diabetes symptoms are often used as criteria for validating in vivo diabetic models. As a result, this model provides a solid foundation for investigating diabetes consequences, such as diabetic osteoporosis and other metabolic bone disorders.

Llabre et al. used an HFD of 46% fat, 36% carbs and 18% protein for 22 weeks to induce diabetic osteoporosis in mice. The HFD-fed mice exhibited an obese phenotype, with significant increases in body mass, blood glucose levels (>250 mg/dL), leptin and insulin-like growth factor-1 (IGF-1) levels. Micro-CT examination revealed changes in bone microarchitecture and enhanced mineralisation, as indicated by higher volumetric bone mineral density (vBMD). Meanwhile, X-ray diffraction (XRD) research corroborated the increase in mineral crystal size in diabetic mice. Mechanical testing presented a reduction in initiation and maximum toughness characteristics, which measure the stress intensity factor at the notch tip and are calculated from yield and ultimate force, respectively. Biochemical studies revealed a significant buildup of advanced glycation end products (AGEs) in bone tissue [13]. The accumulation of AGEs has been linked to decreased bone turnover and fracture resistance by hardening of the organic matrix, altering collagen fibril organisation and affecting fibrillar sliding, consequently limiting energy dissipation inside the bone [17]. Furthermore, increased AGE deposition reduces collagen-bound water content, which contributes to decreased bone toughness and a higher risk of fracture [18].

From the study by Nirwan and Vohora (2022) [14], a high-fat diet (60% kcal from fat) administered for 22 weeks resulted in a marked deterioration in bone quality. Micro-CT analysis demonstrated significantly impaired bone microarchitecture, including reductions in BV/TV, BMD, Tb.N, Tb.Th and Conn.D, along with increased Tb.Sp. Histological assessment revealed thinner, irregular and ruptured bone structures, accompanied by an increased number of osteoclasts and a decrease in osteoblasts and osteocytes. Biochemical findings further supported these observations, with decreased levels of alkaline phosphatase (ALP), bone morphogenetic 2 (BMP2) and osteocalcin, whereas sclerostin and tartrate-resistant acid phosphatase (TRAP) were elevated, indicating reduced osteogenesis and enhanced bone resorption. Consistent with this, immunohistochemical analysis of femoral trabecular bone showed markedly stronger BMP-2 expression in control mice, reflecting active bone formation and healthy bone turnover. In contrast, HFD-fed mice exhibited significantly diminished BMP2 immunoreactivity, which highlights impaired osteogenic signalling and suppressed bone formation under the metabolic stress induced by long-term high-fat-diet feeding.

A recent study by Xing et al. (2025) [15] investigated the effects of an HFD (60% kcal from fat) on bone quality at two timepoints, 4 weeks and 16 weeks, in male C57BL/6J mice. The study showed that HFD feeding caused progressive cancellous bone loss, as demonstrated by significant reductions in BV/TV, BMD and Tb.N, along with increased trabecular separation Tb.Sp at both 4 and 16 weeks. Notably, Tb.Th and cortical parameters, including cortical area (Ct.Ar) and cortical thickness (Ct.Th), remained unchanged, indicating preferential loss of cancellous bone. Molecular and biochemical analyses supported these structural findings. Osteogenic activity was suppressed, reflected by reduced runt-related transcription factor 2 (Runx2) expression and decreased serum levels of procollagen type 1 N-terminal propeptide (P1NP), a marker of bone formation. In contrast, bone resorption was elevated, with increased expression of TRAP and higher circulating levels of C-terminal telopeptide of type I collagen (CTX-1), a biochemical marker of bone degradation. Early inflammatory changes were evident at 4 weeks, including increased F4/80-positive macrophages and elevated expression of tumour necrosis factor-alpha (TNF-α). Transcriptomic profiling revealed time-dependent alterations in biological pathways. Within four weeks, genes related to immune and inflammatory responses were predominantly upregulated. By 16 weeks, pathways associated with extracellular matrix (ECM) organisation, ossification and osteogenic signalling, including the advanced glycation end-products and their receptor (AGE/RAGE) axis, Wnt signalling and the phosphoinositide 3-kinase/protein kinase B (PI3K–Akt pathway) were significantly altered. Together, these findings indicate that prolonged high-fat-diet intake triggers early immune activation followed by impaired matrix regulation and osteogenic signalling, ultimately leading to progressive trabecular bone loss.

Li et al. (2016) [16] investigated the effects of a high-fat diet (60% kcal from fat) administered for 20 weeks in osteoprotegerin (OPG) knockout mice, a model highly susceptible to excessive osteoclast activity and trabecular bone loss. Micro-CT analysis demonstrated substantial deterioration of bone microarchitecture by reduced BV/TV, decreased BMD and lower Tb.N, indicating pronounced compromise of cancellous bone integrity. Western blot analysis further revealed robust activation of multiple osteoclastogenic and inflammatory signalling pathways. High-fat feeding significantly upregulated components of the PI3K/Akt pathway, c-Jun N-terminal kinase/mitogen-activated protein kinase (JNK/MAPK) signalling and nuclear factor-kappa B (NF-κB). Increased phosphorylated Akt (p-Akt) was also observed, which in turn activated the mammalian target of rapamycin (mTOR) pathway, further amplifying downstream metabolic and inflammatory responses. Enhanced receptor activator of nuclear factor-κB ligand (RANKL)–RANK signalling was also detected, accompanied by elevated inflammatory cytokine levels. Upregulation of c-Jun, an essential transcription factor for osteoclast differentiation, supported the increase in osteoclastogenic activity. Taken together, these molecular changes illustrate that long-term high-fat-diet intake in osteoprotegerin (OPG)-deficient mice accelerates trabecular bone loss by promoting metabolic dysregulation, inflammation and excessive osteoclast-mediated bone resorption. The study also provides insight into the broader pathological mechanisms relevant to diabetic osteoporosis. The PI3K/Akt signalling pathway, which mediates the effects of extracellular growth factors and membrane-associated tyrosine kinases, activates downstream regulators such as NF-κB and mTOR. In diabetic osteoporosis, elevated phosphorylated Akt levels have been linked to heightened mTOR activity, thereby intensifying bone loss and disease progression. OPG deficiency further aggravates this process by increasing RANKL–RANK interactions, which boost Akt pathway activation. Activated Akt also elevates phosphorylated NF-κB levels, stimulating inflammatory cytokine production through direct phosphorylation and the NIK/IKK/IκBα cascade, which promotes NF-κB nuclear translocation. These converging pathways synergistically enhance osteoclast differentiation, leading to pronounced trabecular bone loss. Additionally, activation of the MAPK/JNK pathway was shown to contribute to the development of diabetic osteoporosis in this model. JNK phosphorylation elevated c-Jun expression, further promoting osteoclast precursor differentiation and aligning with increased phosphorylated NF-κB levels. Overall, diabetic osteoporosis combined with OPG deficiency markedly activated the PI3K/Akt, JNK/MAPK and NF-κB pathways, upregulated their downstream targets, intensified inflammatory cytokine release and ultimately disrupted trabecular bone architecture, accelerating osteoclast-mediated bone resorption.

### 2.2. Streptozotocin Model of Diabetic Osteoporosis

Streptozotocin (STZ) is a glucosamine-nitrosourea compound produced by Streptomyces achromogenes, originally used as an antibiotic but now widely utilised to induce experimental diabetes in research settings [19]. When administered peripherally via intraperitoneal injection, STZ preferentially destroys pancreatic β cells, resulting in insulin insufficiency, hyperglycaemia, polydipsia and polyuria, the characteristic features of type 1 diabetes [20]. Owing to this selective toxicity, STZ has been extensively employed to establish diabetic osteoporosis models in mice. Importantly, the skeletal alterations induced by STZ closely mirror the bone pathological changes observed in human type 1 diabetes mellitus (T1DM), which further validate its relevance as an experimental model [21,22]. This diabetogenic effect arises because STZ is not only toxic to pancreatic β cells but also to skeletal muscle, as both tissues express high levels of the glucose transporter GLUT2, which facilitates intracellular STZ uptake and subsequently leads to DNA damage [23]. Since skeletal muscle plays a crucial role in regulating bone mass and quality, its vulnerability to STZ toxicity likely contributes to diabetes-associated skeletal deterioration [24]. Supporting this notion, several studies have demonstrated that STZ administration reduces bone formation and increases osteoclast activity. Consistently, other studies reported that STZ decreases bone mineral density (BMD) and induces trabecular bone loss, largely through mechanisms driven by oxidative stress and chronic hyperglycaemia (Table 2).

Wu and Yan (2015) [25] mentioned that STZ-induced diabetic mice treated with 35 mg/kg body weight for five consecutive days exhibited features of diabetic osteoporosis characterised by functional and structural bone impairments. Urinary calcium levels were significantly elevated compared with controls, indicating enhanced calcium excretion due to hyperglycaemia. Further, serum analyses showed increased TRACP-5b and decreased osteocalcin (OCN), reflecting enhanced bone resorption and reduced bone formation. Histological examination of the proximal tibia revealed disrupted connections between the growth plate and trabecular bone, with marked reductions in trabecular bone mass in both the primary and secondary spongiosa. TRAP staining confirmed increased osteoclast numbers, while molecular analyses indicated downregulation of BK1R/BK2R and EphB2/EphrinB2 signalling, contributing to reduced insulin levels and impaired bone remodelling.

Zheng et al. (2017) and Yao et al. (2018) [26,27], both using STZ at 60 mg/kg for five consecutive days, consistently demonstrated that STZ-induced diabetes causes profound deterioration of trabecular bone microarchitecture and accelerates bone resorption. Micro-CT analysis from both studies showed reduced BV/TV and Tb.N, with Zheng et al. reporting increased TbSp, while Yao et al. (2018) [27] also observed reduced Tb.Th and loss of trabecular bone mass histologically. TRAP and IHC staining confirmed a significant increase in osteoclast numbers in their studies. Biochemically, both models exhibited reduced urinary calcium excretion and increased serum ALP and TRAP, together with decreased OPG and increased RANKL, displaying activation of osteoclastogenesis via disruption of the OPG/RANKL balance [26,27]. Yao et al. (2018) further demonstrated broader molecular disturbances: reduced Runx2 signifying impaired osteoblast differentiation; decreased cathepsin K and NFATc1 suggesting altered osteoclast maturation and resorptive function, increased TNF-α indicating heightened inflammatory signalling that suppresses osteoblast activity and promotes osteoclastogenesis [27], elevated caspase-3 reflecting enhanced apoptosis and further compromising bone formation, as well as reductions in phosphorylated AMPK-α and ACC-α indicating disrupted cellular energy metabolism, which weakened osteoblast survival and osteoclast regulation [27,30]. Collectively, these findings highlight that STZ-induced hyperglycaemia drives severe trabecular bone loss by simultaneously increasing osteoclast activity, suppressing osteoblast function, and amplifying inflammation and apoptosis, as well as impairing metabolic signalling essential for bone homeostasis.

Cao et al. (2021) [28] reported that STZ-induced diabetic mice (60 mg/kg body weight for five consecutive days), using a model similar to Zheng et al. and Yao et al., exhibited pronounced trabecular bone loss. Micro-CT analyses showed decreased BV/TV, Tb.Th and Tb.N. Biochemical assessments revealed elevated serum ALP and TRAP, along with decreased OPG and increased RANKL, indicating enhanced osteoclastogenesis through disruption of the RANKL/OPG balance. At the molecular level, increased activation of NF-κB and toll-like receptor 4 (TLR4) suggested that innate immune signalling contributes to heightened inflammation and osteoclast activity under hyperglycaemic conditions. Meanwhile, the activation of TLR4 further led to increased insulin receptor expression and Akt phosphorylation, accompanied by decreased IRS-1 (Ser307) levels. In type 1 diabetes, severe insulin deficiency was associated with loss of TLR4-mediated signalling [31]. Collectively, these insights indicate that STZ-induced diabetes promotes trabecular bone deterioration by simultaneously activating inflammatory pathways, altering insulin signalling and stimulating osteoclast-mediated bone resorption, ultimately impairing overall bone homeostasis.

A single intraperitoneal injection of STZ at 130 mg/kg body weight by Tian et al. (2022) [29] induced diabetic osteoporosis in mice, leading to significant deterioration of trabecular microarchitecture. Micro-CT evaluation showed pronounced reductions in Conn.D, BMD, BV/TV, bone surface density (BS/TV) and Tb.Th, alongside increased trabecular Tb.Sp. These skeletal impairments were accompanied by elevated inflammatory markers, including TNF-α, IL-6, IL-1β and activation of the IKK pathway, indicating that STZ-induced hyperglycaemia promotes bone loss through inflammation-driven disruption of bone remodelling. Network pharmacological analysis further predicted that the STZ model activates the PI3K/Akt signalling pathway, which plays an essential role in the pathogenesis of diabetic osteoporosis. The downstream effector NF-κB, closely linked to glucose and lipid metabolic regulation, was also activated, highlighting the involvement of the PI3K/Akt/NF-κB axis in oxidative stress responses, inflammation and immune regulation [32]. Among these components, Akt functions as a key mediator of bone homeostasis by modulating osteoblast and osteoclast differentiation and suppressing inflammatory cytokine release [33]. In contrast, NF-κB activation enhances inflammatory signalling and promotes osteoclast precursor maturation [34]. The combined activation of PI3K/Akt and NF-κB not only intensified systemic inflammation but also increased TNF-α activity, which is known to inhibit osteoblast differentiation, promote inflammatory cytokine production and induce osteoblast apoptosis, ultimately impairing bone formation and exacerbating STZ-induced diabetic bone loss.

### 2.3. Combined High-Fat Diet and Streptozotocin Model of Diabetic Osteoporosis

A large dose of streptozotocin (STZ; >50 mg/kg) induced hyperglycaemia in mice by selectively destroying pancreatic β cells through DNA alkylation and oxidative stress, resulting in severe insulin deficiency and metabolic characteristics that closely resemble type 1 diabetes mellitus (T1DM) [35]. This single-high-dose STZ model reliably reproduces many hallmark features of T1DM, including rapid onset of hyperglycaemia, weight loss, polyuria and polydipsia, but it is less responsive to interventions targeting insulin resistance, making it unsuitable for evaluating insulin-sensitising or metabolic-modulating therapies [36]. In contrast, the combination of a high-fat diet (HFD) with a low-dose STZ regimen (commonly 25–35 mg/kg) has emerged as a robust and widely used approach to model type 2 diabetes mellitus (T2DM) in rodents. In this two-hit model, prolonged consumption of HFD first induces obesity, peripheral insulin resistance, dyslipidaemia and low-grade systemic inflammation [37]. Subsequent administration of low-dose STZ partially impairs β-cell function without causing complete β-cell destruction. This process produces a metabolic state that closely mirrors human T2DM, characterised by moderate hyperglycaemia, impaired glucose tolerance and hyperinsulinaemia, followed by β-cell exhaustion and chronic metabolic imbalance. Importantly, the HFD + low-dose STZ model also reproduces multi-organ complications associated with T2DM, including hepatic steatosis, renal injury, cardiovascular alterations and skeletal deterioration [38]. Given the ability of the model to maintain insulin resistance and residual β-cell dysfunction, it is particularly suitable for evaluating therapeutic candidates such as insulin sensitisers (such as metformin and TZDs), incretin-based therapies, antioxidants and bone-targeted interventions [39]. In the context of DOP, this model is highly valuable as it exhibits bone microarchitectural deterioration, reduced bone formation and impaired osteoblast function, mirroring key pathological features observed in human T2DM-associated bone fragility (Table 3).

Sihota et al. (2020) [40] developed a non-obese T2DM rat model by administering a high-fat diet containing 58% fat for four weeks, followed by a low dose of streptozotocin (35 mg/kg). The diabetic rats showed significant deterioration in bone quality across multiple hierarchical levels. Micro-CT analysis demonstrated reductions in BMD, BV/TV, Ct.Ar and Ct.Th, indicating compromised microarchitecture. Mechanical testing confirmed a decline in whole-bone strength and increased indentation distance, consistent with weaker and more deformable bones. At the tissue level, nanoindentation revealed a lower modulus, reflecting reduced stiffness. Compositional analysis showed a decreased mineral-to-matrix ratio, while X-ray diffraction indicated larger and broader mineral crystallites, both pointing to disrupted mineral organisation. Biochemically, diabetic bone exhibited elevated non-enzymatic cross-link ratio (NE-xLR) and increased AGE accumulation, signifying substantial collagen glycation. These structural, mechanical and biochemical alterations resulted in greater bone fragility and fracture susceptibility. Fragility in this T2DM model arises from poor glycation control, altered mineralisation quality and compromised collagen–mineral interactions, leading to reduced bone toughness despite normal or even elevated BMD. This model closely mimics the natural progression of late-stage, non-obese T2DM, transitioning from insulin resistance to hypoinsulinaemia. It is particularly relevant for investigating diabetic bone fragility in young or adolescent Asian T2DM populations, where similar metabolic trajectories are commonly observed.

Guo et al. (2019) [41] investigated the effects of a high-fat diet (HFD, 45% fat for four weeks) followed by streptozotocin (STZ, 35 mg/kg for 2 consecutive days) administration on bone health in a diabetic osteoporosis model. Micro-CT and histological analyses revealed significant bone deterioration, including decreased BMD, reduced BV/TV, trabecular thinning and structural impairment of the femur. At the molecular level, bone formation was impaired, as evidenced by reduced levels of BALP, OPG and Runx2, while bone resorption was enhanced, made apparent by increased TRAP activity and TRACP-5b, β-CTX and RANKL expression. Inflammatory markers, including TNF-α, IL-1β, COX-2 and MMP-14, were elevated, suggesting an inflammatory environment contributing to bone loss. Apoptotic signalling was also increased, with upregulation of Caspase-8, -9 and -3, along with elevated levels of pro-apoptotic proteins such as Bad and Bax and decreased anti-apoptotic proteins such as Bcl-2 and Bcl-xl, indicating enhanced osteoblast and osteocyte apoptosis. Importantly, HDAC1 and HDAC3 expression in the femoral head was significantly increased, highlighting a role for epigenetic regulation in diabetic bone pathology. HDAC1/3, which belong to class I histone deacetylases, are critical for cell survival, proliferation and gene expression; overactivity impairs osteoblast function, increases marrow fat with age and is related to inflammation and apoptosis, whereas loss of HDAC3 in osteoprogenitors upregulates osteoprotegerin and improves systemic insulin sensitivity. Overall, these findings suggest that HFD- and STZ-induced diabetic osteoporosis results from a combination of reduced osteogenesis, increased bone resorption, heightened inflammation, elevated apoptosis and aberrant HDAC1/3 activity, collectively contributing to bone fragility.

Zhao et al. (2021) [42] reported that mice fed a high-fat diet containing 55% fat for four weeks, followed by intraperitoneal injection of 0.5% STZ solution, exhibited deterioration of the femoral trabecular microarchitecture, as evidenced by a significant reduction in BMD. Furthermore, the levels of ALP in the femur, thoracic vertebra and lumbar vertebra of diabetic mice were markedly reduced compared to the control group, indicating impaired bone formation under diabetic conditions. In addition, previous studies have shown that this model displays decreased transforming growth factor-β1 (TGF-β1) expression following bone injury compared with baseline levels. Since TGF-β1 is secreted by osteoblasts and plays a key role in promoting osteoblast differentiation while inhibiting osteoclast activity, its downregulation may contribute to the impaired bone remodelling observed in diabetic osteoporosis.

Peng et al. (2021) [43] isolated adipose-derived stem cells from diabetic osteoporosis mice (DOP-ASCs) and later discovered that these cells exhibited decreased DNA methylation levels in the sFrp2 promoter region, along with reduced expression of Wnt signalling pathway markers and diminished osteogenic differentiation potential. The Wnt signalling pathway plays a critical role in stem cell differentiation and development owing to its high evolutionary conservation, structural complexity and ability to integrate signals from multiple developmental cascades. The canonical Wnt/β-catenin pathway and noncanonical pathways (Wnt/PCP and Wnt/Ca^2+^) regulate bone modelling and remodelling by modulating energy metabolism and osteogenic activity in osteoblasts. Secreted frizzled-related protein 2 (sFrp2) functions as an antagonist of the canonical Wnt pathway by binding to Wnt ligands via its cysteine-rich or C-terminal netrin-like domains, or by forming nonfunctional complexes with frizzled receptors, thereby inhibiting Wnt signalling. As a key regulator within this pathway, sFrp2 influences stem cell proliferation, apoptosis and differentiation by modulating Wnt activity and is thus involved in multiple biological processes. In summary, the study demonstrated that the impaired osteogenic potential of DOP-ASCs may be attributed to altered Wnt signalling regulation through DNA methylation changes in the sFrp2 promoter region.

Zhang et al. (2024) [44] established a diabetic osteoporosis (DOP) rat model by feeding rats a high-sugar, high-fat diet (31.1% fat, 53.3% sugar) for four weeks before administering intraperitoneal STZ injections (35 mg/kg for two consecutive days). Immunohistochemical examination of femoral tissue indicated a significant drop in Osteoglycin (OGN) and Runx2 expression, as well as trabecular bone loss. OGN regulates osteoblast activity, insulin action and glucose metabolism. Reduced OGN expression impairs osteogenic signalling and is linked to poor bone formation in DOP. As a tiny leucine-rich proteoglycan, OGN stimulates mineralisation and has a favourable impact on bone development [44]. Lower serum OGN levels have been linked to decreased bone mineral density (BMD) and an increased risk of vertebral fractures in postmenopausal women with type 2 diabetes [47]. Aside from its skeletal effects, OGN is associated with metabolic regulation. Evidence suggests that OGN regulates systemic glucose homeostasis via a muscle–pancreas–bone axis. Exogenous OGN treatment lowered blood glucose in a dose-dependent manner and boosted insulin’s glucose-lowering effects in glucose-tolerance trials in rats, showing that OGN may improve insulin sensitivity [48]. As a result, decreased OGN expression in diabetic osteoporosis may suppress osteogenic factor production, including Runx2, compromising osteoblast activity and contributing to DOP pathology.

Hyperglycaemia enhances the generation of reactive oxygen species (ROS), disrupting the balance between oxidants and antioxidants and contributing to inflammation-related complications like diabetic osteoporosis [49]. Since excess ROS and subsequent oxidative damage are major drivers of diabetes-induced tissue dysfunction, there is a need for therapeutic strategies that target oxidative stress. In bones, ROS promotes diabetic osteoporosis by increasing osteoclastic bone resorption while suppressing osteoblast development and activity [50]. Apparently, antioxidants can mitigate these effects not just by directly scavenging ROS, but also by preserving intracellular redox balance and activating endogenous defence systems. Among these regulators, nuclear factor erythroid 2-related factor 2 (Nrf2) acts as a master transcription factor that controls antioxidant gene expression, protects the bone marrow microenvironment from oxidative damage and promotes the activity of bone marrow stem cells [51]. Increasing data suggest that Nrf2 and its downstream pathways are critical for preventing diabetic complications, including diabetic bone loss [52]. Consistent with this, Wang et al. (2024) [45] found that rats fed with a high-fat, high-sucrose diet for four weeks followed by STZ (30 mg/kg) had conventional osteoporotic characteristics, such as decreased BMD, BV/TV and trabecular thickness, as well as increased trabecular spacing. Histological examination revealed trabecular depletion, thinning, disintegration and decreased new bone formation, as well as increased bone marrow adiposity. Meanwhile, molecular indicators displayed poor bone metabolism, with elevated TRAP5b and PINP, decreased Runx2, and upregulated PPARγ, as well as significantly reduced Nrf2 and HO-1 expression, highlighting the essential role of oxidative stress and Nrf2 suppression in diabetic osteoporosis.

In addition, recent research suggests that Tissue Inhibitor of Metalloproteinases-1 (TIMP1) may serve as a potential biomarker and therapeutic target associated with ferroptosis [53,54]. Peng et al. (2024) [46] investigated the molecular function of TIMP1 in diabetic osteoporosis (DOP). A mouse model of DOP was established using a high-fat diet combined with low-dose STZ administered over 4 consecutive days, as described in Peng et al. (2024) [46], providing the minimum information necessary for reproducibility. Using this model, the authors demonstrated that TIMP1 interacts with TFRC to regulate iron uptake and ferroptosis in osteoblasts. Knockdown of TIMP1 reduced ferroptosis by increasing GPX4 expression, leading to improved bone microarchitecture and osteoblast function. To specifically reduce TIMP1 expression, mice received injections of lentivirus carrying shRNA targeting TIMP1 (LVshTIMP1), while control mice received a non-targeting lentivirus (LV-NC). Micro-CT imaging revealed a significant reduction in bone volume in HFD + STZ mice compared to controls, along with decreases in BMD, BV/TV and Tb.N. Immunohistochemical examination revealed that TIMP1 knockdown drastically reduced TIMP1 and TFRC, while considerably increasing Glutathione peroxidase 4 (GPX4) expression. Western blot analysis indicated a significant rise in GPX4 levels after TIMP1 knockdown. Overall, these results suggest that TIMP1 knockdown can reduce ferroptosis in vivo and improve diabetic osteoporosis. Transferrin receptor (TFRC) is known to increase intracellular iron levels by facilitating iron uptake, which promotes reactive oxygen species (ROS) formation and lipid peroxidation [55]. TIMP1, a secreted protein known for its inhibitory activity on matrix metalloproteinase-9 (MMP9), has not been thoroughly investigated for its mechanistic role in diabetic osteoporosis [56]. Peng et al. (2024) [46] provides strong evidence that ferroptosis causes osteoblast death in vivo in diabetic osteoporosis. TIMP1 was discovered to interact with TFRC in diabetic circumstances, resulting in increased TFRC expression and osteoblast sensitivity to ferroptosis. Furthermore, pharmacological suppression of ferroptosis significantly reduced osteoblast mortality and trabecular bone degradation.

## 3. Interaction Between Diabetes and Bone

**Figure 1:**
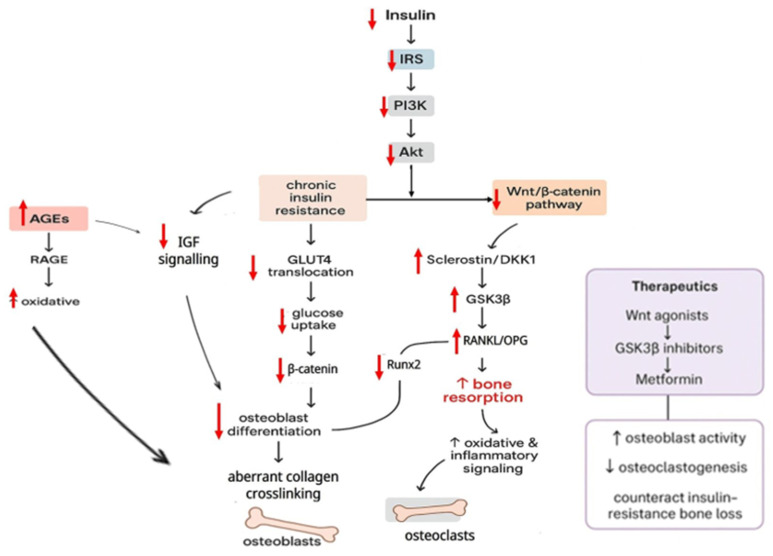
Illustrates insulin and IGF-I signalling in bone metabolism. Insulin and IGF-I control osteoblast proliferation, differentiation and matrix synthesis through PI3K/Akt, Wnt/β-catenin, and RANK/RANKL/OPG pathways. Diabetes and insulin resistance disrupt these signals, boosting Wnt inhibitors (sclerostin, DKK1), activating GSK3β and promoting AGE buildup, resulting in reduced bone growth and fragility. Therapeutic interventions aimed at these pathways can restore osteoblast function and enhance bone health. Upward arrows (↑) indicate increased, and downward arrows (↓) indicate decreased.

Insulin and its associated signalling pathways play a critical role in bone metabolism, particularly in the processes of osteoblastic differentiation, collagen synthesis and overall bone formation (Figure 1). T1DM and T2DM are linked with substantial reductions in BMD, impaired bone formation and an elevated risk of fragility fractures. These clinical observations strongly indicate that insulin signalling is essential for maintaining skeletal development and bone homeostasis [57,58,59]. Osteoblasts, the primary bone-forming cells, express functional insulin receptors, while insulin has been shown to stimulate their proliferation and differentiation. Studies employing osteoblast-specific insulin receptor knockout mice (Ob-IR-/-) reported significant reductions in bone volume due to decreased osteoblast numbers and impaired bone formation. In these mice, ALP activity and osteocalcin expression were markedly reduced, partially as a result of increased Twist2 expression, a known inhibitor of Runx2, underscoring insulin’s role in promoting osteogenic transcriptional activity [60,61].

Beyond its involvement in bone formation, IGF-I also contributes to maintaining the equilibrium between osteoblastic bone formation and osteoclastic resorption. Clinical research consistently recorded strong associations between IGF-I levels and indices of bone density [62]. In metabolic conditions characterised by insulin resistance, such as obesity and diabetes, osteoblast function becomes compromised partly due to the suppression of Wnt/β-catenin signalling [63]. This suppression enhances the production of Wnt inhibitors, notably sclerostin. Elevated sclerostin concentrations documented in individuals with obesity, impaired glucose tolerance and both major types of diabetes reflect this disruption [64]. Importantly, short-term increases in circulating insulin do not appear to alter sclerostin levels, suggesting chronic insulin resistance and broader metabolic abnormalities as the primary drivers [65]. Collectively, these observations indicate that disturbances in IGF-I signalling, combined with altered Wnt pathway activity, reduce osteoblast differentiation and contribute to the diminished bone quality observed in diabetic populations.

IGF-I and its receptor are also intimately connected to glucose handling, insulin biosynthesis and skeletal maturation. In a study by Ardawi and colleagues (2013), postmenopausal women with type 2 diabetes who had suffered vertebral fractures exhibited notably higher circulating sclerostin concentrations, increased markers of bone resorption such as CTX and reduced formation markers, including osteocalcin and P1NP [66]. Lower levels of circulating IGF-I in these individuals were strongly associated with fracture occurrence, underscoring the importance of intact IGF-I signalling for maintaining vertebral strength [67]. Chronic hyperglycaemia characteristic of diabetes accelerates the formation of advanced glycation end products (AGEs), which arise from non-enzymatic interactions between reducing sugars and biological macromolecules. As AGEs accumulate within bone matrix collagen, they intensify cross-link formation, increase matrix brittleness and diminish mechanical resilience. In addition to these structural consequences, AGEs interact with the receptor for advanced glycation end products (RAGE) on osteoblasts and progenitor cells, triggering oxidative and inflammatory signalling cascades. These responses interfere with IGF-I receptor activation and downstream pathways that support osteoblast differentiation. The resulting reduction in osteoblast responsiveness to IGF-I contributes to bone fragility commonly observed in individuals with diabetes [68].

Pancreatic β cells regulate insulin secretion by assessing glucose, amino acid and lipid levels. Insulin maintains overall glucose homeostasis primarily by increasing glucose absorption in skeletal muscle and adipose tissue via GLUT4 mobilisation to the cell surface. When insulin binds to its receptor, conformational changes in the α-subunits allow for autophosphorylation, resulting in the recruitment and phosphorylation of insulin receptor substrates (IRSs). These IRSs activate PI3K, which converts PIP2 to PIP3, allowing Akt to be further activated downstream. Akt then promotes the translocation of GLUT4 to the plasma membrane, which boosts cellular glucose absorption. Akt also inhibits the action of GSK3β, which promotes glycogen synthesis and contributes to metabolic stability. Since key metabolic pathways overlap with skeletal signalling networks, insulin’s actions extend to regulating osteoblast survival, maturation and extracellular matrix deposition [69]. Insufficient insulin production or peripheral insulin resistance disrupts insulin signalling, affecting PI3K/Akt, canonical Wnt/β-catenin and the RANK/RANKL/OPG axis. These pathways are important for bone physiology. Reduced insulin availability inhibits PI3K/Akt activity, lowering osteoblast viability, Runx2 expression, glucose uptake by bone-forming cells and matrix synthesis. Under normal settings, PI3K/Akt activation can boost OPG expression while suppressing RANKL, limiting osteoclast growth and promoting net bone formation [70]. Insufficient insulin disrupts Wnt/β-catenin signalling, resulting in increased synthesis of Wnt inhibitors such as sclerostin and DKK1, which activate GSK3β and accelerate β-catenin destruction. As a result, osteoblast development diminishes, mesenchymal stem cells transition to adipogenic fates and marrow adiposity rises, all of which degrade bone integrity. As GSK3β lies at the intersection of PI3K/Akt and Wnt pathways, disturbances in either pathway affect β-catenin-dependent regulation of the OPG/RANKL ratio. In insulin-deficient states, reduced OPG levels facilitate osteoclastogenesis and heighten bone resorption [71].

Therapeutic drugs targeting common pathways, such as metformin, Wnt pathway agonists and GSK3β inhibitors, can increase osteoblast activity and decrease osteoclast development [72,73,74]. Continuous insulin exposure can restore the inhibition of Akt and GSK3β phosphorylation induced by insulin shortage, highlighting insulin’s direct impact on bone-related signalling pathways. Additional metabolic stressors, including chronic hyperglycaemia and the accumulation of advanced glycation end products (AGEs), exacerbate osteoblast dysfunction by increasing oxidative stress, activating receptors for AGE (RAGE) signalling and reducing insulin-like growth factor-I (IGF-I) sensitivity (Figure 1). Clinical evaluation of postmenopausal women with type 2 diabetes mellitus (T2DM) has demonstrated that a lower bone material strength index is significantly associated with increased AGE accumulation, as assessed by skin autofluorescence [75]. Moreover, recent clinical studies have shown that patients with uncontrolled T2DM exhibit significantly elevated levels of AGEs and RAGE compared with non-diabetic controls, with a strong positive correlation observed between RAGE expression and HbA1c levels (*p* < 0.01) [76]. Chronic hyperglycaemia further disrupts osteoblast function by inhibiting protein kinase C activity, increasing flux through the polyol pathway and impairing multiple growth factor signalling pathways, collectively restricting osteoblast proliferation, differentiation and extracellular matrix synthesis [77]. Given that AGE accumulation consistently predicts fracture risk in diabetic patients, the assessment of AGEs may offer clinical value in the individualised management of diabetes-associated bone fragility. These molecular disturbances may occur before changes in standard biochemical markers of bone turnover are detectable, suggesting that diabetic bone disease often begins at a subclinical level.

## 4. Conclusions and Future Perspective

The current in vivo models of diabetic osteoporosis, which included the high-fat-diet (HFD), streptozotocin (STZ) and combined HFD + STZ protocols, provided compelling evidence that chronic metabolic dysfunction causes significant and multifactorial skeletal degradation. Micro-CT, histomorphometry, biomechanical testing and biochemical analyses consistently showed significant losses in trabecular and cortical bone mass, decreased bone mineral density, compromised structural connectivity and impaired mechanical properties in these models, indicating clinically observed diabetic bone fragility. Obesity-driven insulin resistance was seen as the primary cause of HFD-induced diabetes. Meanwhile, chronic hyperinsulinemia, dyslipidaemia, oxidative stress and low-grade inflammation degraded osteoblast development and function, altered Wnt/β-catenin signalling and promoted osteoclastogenesis. In contrast, STZ-induced diabetes replicated the insulin-deficient state of type 1 diabetes, where selective pancreatic β-cell death caused severe hyperglycaemia and increased oxidative stress and inflammation, inhibiting bone formation and accelerating resorption. Although streptozotocin (STZ) is widely used to induce experimental diabetes in mice, it has several important limitations. In addition to its dose-dependent β-cell toxicity, STZ is associated with off-target effects, including nephrotoxicity, hepatotoxicity and generalised systemic stress. Moreover, variability in responses to STZ across mouse strains, ages, sexes and routes of administration can lead to inconsistent induction of hyperglycaemia. High-dose STZ regimens are also associated with increased systemic toxicity and mortality [78]. Collectively, these limitations underscore the need for careful model design, rigorous standardisation and cautious interpretation of findings when using STZ-based models in diabetes research.

The HFD + STZ paradigm combined peripheral insulin resistance with partial β-cell malfunction, leading to severe hyperglycaemia, systemic inflammation, lipid abnormalities and increased oxidative stress. This dual-hit model closely mirrors human type 2 diabetes, exhibiting extensive trabecular bone loss, impaired cortical microarchitecture, collagen crosslinking abnormalities and dysregulated osteoblast–osteoclast signalling, making it a robust translational platform for preclinical evaluation of therapeutic interventions targeting diabetic bone fragility. Collectively, these models provide invaluable mechanistic insights and facilitate the development of strategies to mitigate diabetes-associated skeletal deterioration.

Overall, these models’ findings showed that diabetic osteoporosis is caused by a complex interplay of endocrine disturbances, oxidative damage, impaired insulin/IGF-I signalling, osteocyte dysfunction and qualitative changes in the bone matrix, including increased AGE-mediated collagen crosslinking. These mechanisms damage the cellular and extracellular components of bones, reducing bone quality regardless of mineral density. Future research should focus on determining the contributions of AGE-RAGE signalling, mitochondrial dysfunction, autophagy dysregulation, gut microbiota changes and bone–fat–muscle crosstalk to diabetic skeletal degeneration. Most in vivo studies use male rodents, which may limit generalizability due to sexual dimorphism in bone metabolism and diabetes-related skeletal outcomes. Female rodents differ in bone turnover and hormonal regulation, affecting susceptibility to diabetic bone loss. Additionally, the age at diabetes induction influences bone remodelling responses [79]. These factors are important when translating findings to humans, where both sex and age affect diabetic osteoporosis risk and progression. Standardising experimental techniques, including sex-specific analysis and adding multi-omics methodologies (transcriptomics, metabolomics, and lipidomics), will significantly improve the interpretability and translational value of preclinical results. Furthermore, expanding therapeutic investigations beyond anti-diabetic drugs to include bone-targeted antioxidant therapies, osteocyte-directed agents, modulators of Wnt signalling and AGE inhibitors may offer new avenues for preventing or reversing diabetic bone fragility. Together, these future directions will strengthen the bridge between mechanistic discoveries and clinical strategies aimed at improving skeletal health in individuals with diabetes.

## Figures and Tables

**Table 1:** Effects of high-fat-diet-induced diabetic osteoporosis on bone microarchitecture and cellular findings.

Study	Diet and Duration	Bone Structural and Mechanical Outcomes	Key Molecular/Cellular Findings
Llabre et al. [13]	HFD 46% fat/36% carbs/18% protein—22 weeks	Micro-CT: altered microarchitecture; ↑ vBMD. XRD: ↑ mineral crystal size. Mechanical: ↓ initiation and max toughness	↑ AGE accumulation in bone → collagen crosslinking, ↓ collagen-bound water
Nirwan and Vohora [14]	HFD 60% kcal fat, 25% protein and 17% carbohydrate—22 weeks	Micro-CT: altered microarchitecture; ↓ BV/TV, ↓ BMD, ↓ Tb.N, ↓ Tb. No, ↓ Tb.Th, ↓ Conn.D, ↑ Tb.Sp Bone histology: thinner, irregular and ruptured appearance of bone, ↑ osteoclast number, ↓ osteoblast and osteocyte number	↓ ALP, ↓ BMP-2, ↓ osteocalcin, ↑ sclerostin, ↑ TRAP → reduced osteogenesis and increased bone resorption
Xing et al. [15]	HFD 60 kcal% fat—4-week and 16-week timepoints	Micro-CT: 4 and 16 wk: ↓ BV/TV, ↓ BMD, ↓ Tb.N, ↑ Tb.Sp; no change in Tb.Th, Ct.Ar, Ct.Th (cancellous preferential loss)	↓ Runx2, ↑ TRAP; early ↑ F4/80 and ↑ TNF-α; serum ↓ P1NP, ↑ CTX1; transcriptomics: 4 w → immune genes; 16 w → ECM/ossification, AGE-RAGE, Wnt, PI3K-Akt
Li et al. [16]	HFD 60 kcal% fat in OPG knockout mice, 20 weeks	Micro-CT: ↓ BV/TV, ↓ BMD, ↓ Tb.N	↑ PI3K/AKT, ↑JNK/MAPK, ↑ NF-κB; ↑ p-AKT → mTOR; enhanced RANKL–RANK signalling; ↑ inflammatory cytokines; ↑ c-Jun → ↑ osteoclastogenesis

Abbreviation: HFD: High-fat diet; BV/TV: Bone volume/total volume; BMD: Bone mineral density; Tb.N: Trabecular number; Tb.Th: Trabecular thickness; Tb.Sp: Trabecular separation; Conn.D: Connectivity density; AGE: Advanced glycation end products; ALP: Alkaline phosphatase; BMP-2: Bone Morphogenetic Protein-2; TRAP: Tartrate-Resistant Acid Phosphatase; Runx2: Runt-related Transcription Factor 2; F4/80: Macrophage Marker; TNF-α: Tumour Necrosis Factor-alpha; P1NP: Procollagen Type 1 N-terminal Propeptide; CTX1: C-terminal Telopeptide of Type 1 Collagen; PI3K: Phosphoinositide 3-Kinase; AKT: Protein Kinase B; JNK: c-Jun N-terminal Kinase; MAPK: Mitogen-Activated Protein Kinase; NF-κB: Nuclear Factor kappa-light-chain-enhancer of activated B cells; RANKL: Receptor Activator of Nuclear Factor κB Ligand; OPG: Osteoprotegerin; ECM: Extracellular matrix; ↑: increased; ↓: decreased.

**Table 2:** Effects of streptozotocin-induced diabetic osteoporosis on bone microarchitecture and cellular findings.

Study	STZ Dose and Duration	Bone Structural and Mechanical Outcomes	Key Molecular/Cellular Findings
Wu et al. [25]	STZ: 35 mg/kg body weight; 5 consecutive days	Histology: ↓ trabecular bone mass (primary and secondary spongiosa); TRAP staining: ↑ osteoclast number	↓ BK1R/BK2R, ↓ EphB2/EphrinB2, ↓ insulin levels
Zheng et al. [26]	STZ: 60 mg/kg body weight; 5 consecutive days	Micro-CT: ↓ BV/TV, ↓ Tb.N, ↑ Tb.Sp; TRAP staining: ↑ osteoclast number	↓ urinary calcium; ↑ ALP, ↑ TRAP, ↓ OPG, ↑ RANKL → ↑ osteoclastogenesis
Yao et al. [27]	STZ: 60 mg/kg body weight; 5 consecutive days	Micro-CT: ↓ BV/TV, ↓ Tb.Th, ↓ Tb.N; Histology: ↓ trabecular bone mass; IHC: ↑ osteoclast number	↓ urinary calcium; ↑ ALP, ↑ TRAP, ↓ OPG, ↑ RANKL; ↓ Runx2, ↓ Cathepsin K, ↓ NFATc1; ↑ TNF-α, ↑ Caspase 3; ↓ phosphorylated AMPK-α, ↓ ACC-α
Cao et al. [28]	STZ: 60 mg/kg body weight; 5 consecutive days	Micro-CT: ↓ BV/TV, ↓ Tb.Th, ↓ Tb.N	↑ ALP, ↑ TRAP, ↓ OPG, ↑ RANKL; ↑ NF-κB; ↑ TLR4
Tian et al. [29]	STZ: 130 mg/kg body weight, once	Micro-CT: ↓ Conn.D, ↓ BMD, ↓ BV/TV, ↓ BS/TV, ↓ Tb.Th, ↑ Tb.Sp	↑ TNF-α, ↑ IKK, ↑ IL-6, ↑ IL-1β → ↑ inflammation

Abbreviations: STZ: Streptozotocin; BV/TV: Bone volume/total volume; Tb.Th: Trabecular thickness; Tb.N: Trabecular number; Tb.Sp: Trabecular separation; Conn.D: Connectivity density; BMD: Bone mineral density; BS/TV: Bone surface/total volume; TRAP: Tartrate-resistant acid phosphatase; OPG: Osteoprotegerin; RANKL: Receptor activator of nuclear factor kappa-B ligand; ALP: Alkaline phosphatase; IHC: Immunohistochemistry; TNF-α: Tumour necrosis factor alpha; IL-6: Interleukin 6; IL-1β: Interleukin 1 beta; NF-κB: Nuclear factor kappa B; TLR4: Toll-like receptor 4; Runx2: Runt-related transcription factor 2; Cathepsin K: Lysosomal protease involved in bone resorption; NFATc1: Nuclear factor of activated T-cells, cytoplasmic 1; AMPK-α: AMP-activated protein kinase alpha; ACC-α: Acetyl-CoA carboxylase alpha; BK1R/BK2R: Bradykinin receptor 1/receptor 2; EphB2/EphrinB2: Receptor–ligand pair involved in osteoblast–osteoclast communication; ↑: increased; ↓: decreased.

**Table 3:** Effects of combined high-fat diet and streptozotocin-induced diabetic osteoporosis on bone microarchitecture and cellular findings.

Study/Model	Diet/STZ Dose and Duration	Bone Structural and Mechanical Outcomes	Key Molecular/Cellular Findings
Sihota et al. [40]	HFD 58% fat, 4 wk → STZ 35 mg/kg, single dose	Micro-CT: ↓ BMD, ↓ BV/TV, ↓ Ct.Ar, ↓ Ct.Th; Mechanical: ↓ whole-bone strength, ↑ indentation distance; Nanoindentation: ↓ modulus; Bone composition: ↓ mineral/matrix ratio; XRD: ↑ mineral crystallite size	↑ NE-xLR, ↑ AGE → ↑ collagen glycation → ↑ fracture risk
Guo et al. [41]	HFD 45% fat, 4 wk → STZ 35 mg/kg, 2 consecutive days	Micro-CT: ↓ BMD, ↓ BV/TV; Histology: trabecular thinning, structural impairment	↓ BALP, ↓ OPG, ↓ Runx2, ↑ TRAP, ↑ TRACP-5b, ↑ β-CTX, ↑ RANKL → ↓ osteogenesis, ↑ resorption; ↑ TNF-α, ↑ IL-1β, ↑ COX-2, ↑ MMP-14 → ↑ inflammation; ↑ Caspase-8/-9/-3, ↓ Bcl-2/Bcl-xl, ↑ Bad/Bax → ↑ apoptosis; ↑ HDAC1/3 → ↑ inflammation/apoptosis
Zhao et al. [42]	HFD 55% fat, 4 wk → STZ 0.5%, single or multiple dose not specified	↓ BMD; deteriorated femoral trabecular microarchitecture; ↓ ALP (femur, thoracic/lumbar vertebrae)	↓ TGF-β1 → impaired osteoblast differentiation; ↓ ALP → ↓ bone formation
Peng et al. [43]	HFD + low-dose STZ → DOP mice, isolated DOP-ASC	Impaired osteogenic differentiation in DOP-ASCs	↓ sFrp2 promoter DNA methylation; ↓ Wnt/β-catenin signalling → ↓ osteogenic markers; impaired stem cell differentiation
Zhang et al. [44]	High-sugar/high-fat diet (31.1% fat, 53.3% sugar) 4 wk → STZ 35 mg/kg, 2 consecutive days	Micro-CT: ↓ BMD, ↓ BV/TV, ↓ Tb.Th; IHC: ↓ OGN and Runx2; trabecular bone loss	↓ OGN → impaired osteoblast function; ↓ osteogenic factor expression; OGN regulates insulin and glucose metabolism
Wang et al. [45]	65% standard chow + 10% lard, 20% sucrose, 2.5% cholesterol, 1% sodium cholate, 1% mineral mix, 0.5% cellulose, 4 wk → STZ 30 mg/kg, single dose	Micro-CT: ↓ BMD, ↓ BV/TV, ↓ Tb.Th, ↑ Tb.Sp; Histology: trabecular thinning and disorganisation, ↓ new bone formation; ↑ marrow adipocyte density/volume	↑ TRACP-5b, ↑ PINP, ↓ Runx2, ↑ PPARγ, ↓ Nrf2, ↓ HO-1
Peng et al. [46]	HFD + STZ + TIMP1 shRNA KD	↓ BMD, BV/TV, Tb.N in HFD + STZ; TIMP1 KD improves bone	TIMP1 interacts with TFRC → ↑ iron uptake → ↑ ROS → ferroptosis; TIMP1 KD ↑ GPX4 → ↓ ferroptosis

Abbreviation: HFD: High-fat diet; STZ: Streptozotocin; BMD: Bone mineral density; BV/TV: Bone volume/total volume; Ct.Ar: Cortical area; Ct.Th: Cortical thickness; Tb.Th: Trabecular thickness; Tb.N: Trabecular number; Tb.Sp: Trabecular separation; NE-xLR: Non-enzymatic cross-link ratio; AGE: Advanced glycation end products; BALP: Bone-specific alkaline phosphatase; OPG: Osteoprotegerin; Runx2: Runt-related transcription factor 2; TRAP: Tartrate-resistant acid phosphatase; TRACP-5b: tartrate-resistant acid phosphatase 5b; β-CTX: Beta C-terminal telopeptide; RANKL: Receptor activator of nuclear factor kappa-B ligand; ALP: Alkaline phosphatase; IHC: Immunohistochemistry; OGN: Osteoglycin; DOP-ASC: Diabetic osteoporosis adipose-derived stem cells; KD: Knockdown; TFRC: Transferrin receptor; ROS: Reactive oxygen species; GPX4: Glutathione peroxidase 4; PINP: Procollagen type I N-terminal propeptide; PPARγ: Peroxisome proliferator-activated receptor gamma; Nrf2: Nuclear factor erythroid 2-related factor 2; HO-1: Heme oxygenase-1; ↑: increased; ↓: decreased.

## Data Availability

No new data were created or analyzed in this study. Data sharing is not applicable to this article.

## Abbreviations

ACC-α	Acetyl-CoA Carboxylase Alpha
AGE	Advanced Glycation End Products
ALP	Alkaline Phosphatase
AMPK-α	AMP-Activated Protein Kinase Alpha
BALP	Bone-Specific Alkaline Phosphatase
BMD	Bone Mineral Density
BK1R/BK2R	Bradykinin Receptor 1/Receptor 2
BMP-2	Bone Morphogenetic Protein-2
BS/TV	Bone Surface/Total Volume
BV/TV	Bone Volume/Total Volume
Cathepsin K	Lysosomal Protease in Bone Resorption
Conn.D	Connectivity Density
Ct.Ar	Cortical Area
Ct.Th	Cortical Thickness
CTX-1/β-CTX	C-Terminal Telopeptide of Type I Collagen
DOP-ASC	Diabetic Osteoporosis Adipose-derived Stem Cells
EphB2/EphrinB2	Osteoblast–Osteoclast Signalling Pair
ECM	Extracellular Matrix
F4/80	Macrophage Marker
GPX4	Glutathione Peroxidase 4
HFD	High-Fat Diet
HO-1	Heme Oxygenase-1
IHC	Immunohistochemistry
IL-1β	Interleukin-1 Beta
IL-6	Interleukin-6
JNK	c-Jun N-Terminal Kinase
KD	Knockdown
MAPK	Mitogen-Activated Protein Kinase
NFATc1	Nuclear Factor of Activated T Cells, Cytoplasmic 1
NF-κB	Nuclear Factor kappa B
NE-xLR	Non-Enzymatic Cross-Link Ratio
Nrf2	Nuclear Factor Erythroid 2-Related Factor 2
OGN	Osteoglycin
OPG	Osteoprotegerin
P1NP/PINP	Procollagen Type I N-Terminal Propeptide
PI3K	Phosphoinositide 3-Kinase
PPARγ	Peroxisome Proliferator-Activated Receptor Gamma
RANKL	Receptor Activator of Nuclear Factor κB Ligand
ROS	Reactive Oxygen Species
Runx2	Runt-Related Transcription Factor 2
STZ	Streptozotocin
Tb.N	Trabecular Number
Tb.Sp	Trabecular Separation
Tb.Th	Trabecular Thickness
TFRC	Transferrin Receptor
TLR4	Toll-Like Receptor 4
TNF-α	Tumour Necrosis Factor Alpha
TRAP/TRACP-5b	Tartrate-Resistant Acid Phosphatase/Isoform 5b
